# Why does mental health care not follow the structuring of primary care?

**DOI:** 10.11606/s1518-8787.2021055003344

**Published:** 2021-11-23

**Authors:** Antonio Moacir de Jesus Lima, Eli Iola Gurgel Andrade, Antonio Thomaz Gonzaga da Matta Machado, Alainer de Fátima dos Santos

**Affiliations:** I Universidade Federal dos Vales do Jequitinhonha e Mucuri Faculdade de Ciências Biológicas e da Saúde Departamento de Enfermagem Diamantina MG Brasil Universidade Federal dos Vales do Jequitinhonha e Mucuri. Faculdade de Ciências Biológicas e da Saúde. Departamento de Enfermagem. Diamantina, MG, Brasil; II Universidade Federal de Minas Gerais Faculdade de Medicina Belo Horizonte MG Brasil Universidade Federal de Minas Gerais. Faculdade de Medicina. Programa de Pós-Graduação em Saúde Pública. Belo Horizonte, MG, Brasil; III Universidade Federal de Minas Gerais Faculdade de Medicina Departamento de Medicina Preventiva e Social Belo Horizonte MG Brasil Universidade Federal de Minas Gerais. Faculdade de Medicina. Departamento de Medicina Preventiva e Social. Belo Horizonte, MG, Brasil

**Keywords:** Mental Health Assistance, Primary Health Care, Quality of Health Care, Health Services Administration.

## Abstract

**OBJECTIVE:**

To verify if primary care teams with better structured primary health care (PHC) attributes could offer better mental health (MH) care.

**METHODS:**

Cross-sectional study based on data from the external evaluation of the second cycle of the *Programa de Melhoria do Acesso e da Qualidade da Atenção Básica* (PMAQ-AB - Access and Quality Improvement of Primary Care Program), involving 31,587 primary care teams, between 2013 and 2014. Two typologies were built: quality of mental health care (dependent variable) and PHC structuring according to essential attributes (independent variable). We identified some contents for the construction of the mental health typology (module II of the PMAQ) and performed sums of questions for the categorization of indices. The Delphi technique rendered consensus in four rounds endorsed by experts, following the attributes of PHC structure. Multinomial logistic regression analyses verified the association between the typologies and identified which attribute most contributed to the quality of mental health care.

**RESULTS:**

We found out that 29.2% of the teams are at low levels of quality in assistance to MH, while 7.5% of the teams have a low level of structuring the PHC according to essential attributes. Regional differences are maintained, both for the structuring of the PHC and for the quality of mental health care. There was a greater chance of providing care in MH with better quality when the PHC is better structured at a high level (OR = 14.74) and at a medium level (OR = 2.193). A high level of completeness is associated with a high level of Quality of Care in MH (OR = 3.21).

**CONCLUSION:**

results indicate a predominance of low levels of quality in mental health care, out of step with the process of PHC structuring and its essential attributes.

## INTRODUCTION

Mental disorders represent a high share of disease records globally. Only in the Americas, a study^1^ revealed a prevalence of 17%, being 22.5% in North America and 14.8% in Latin America. In Brazil, mental disorders also present a considerable 29.5% rate. Anxiety (19.9%) and mood disorders (11%) are most prevalent, followed by impulse control disorders (4.2%) and others, resulting from the use of psychoactive substances (3.6%). These numbers show that Brazil jumped from sixth to the third position^2^ in the Global Burden of Disease (GBD) between 1990 and 2015.

Screening for early detection and treatment performed in primary health care (PHC) may improve the life of patients, help make better use of investments in health care, and significantly reduce complications and medical comorbidities^3^. Thus, mental health (MH) should be understood as a priority intervention field for the Family Health Strategy (FHS) teams, given the close work in the community, wherein users live and circulate, for example in health units and social space.

However, the inclusion of mental health in PHC practices is difficulty in some aspects, as shown by several studies. One of these obstacles is in professional training because workers developed their actions based on the biological model. Therefore, interventions focused on clinical consultation, medicalization, and diagnosis, aiming only at the remission of symptoms^4^. Other studies reinforce this perception and point to the absence of co-responsibility for unnecessary referrals and an important weakness in the ability of FHS professionals to identify some cases^5,6^. More recently, another study showed that more than 60% of PHC professionals said to feel unprepared to accept mental health demands^7^.

Another obstacle is the work process of teams, considering the humanization of people with psychological distress has not yet been identified, although some practices are innovative^8^. However, this process is still in the implementation phase and, therefore, needs to be expanded through the improvement of care^5^.

This expansion of access also involves issues of infrastructure, both in basic units and in human resources, and the breakdown of the care network, which still maintains a hierarchical organization and communicational dynamics based on technical health protocols^9^. In Salvador, Bahia, for example, a study indicated a dependence on the *Rede de Atenção Psicossocial* (RAPS - Psychosocial Care Network) to the psychiatric hospital, pointing out the need for actions that promote the strengthening of the network, its articulation, and deinstitutionalization of the *Centro de Atenção Psicossocial* (CAPS - Psychosocial Care Center)^10^. In Fortaleza, Ceará, the medicalization of mental disorders was evidenced as mitigation of difficulties in front of CAPS accessibility by patients^11^.

However, the engagement of workers has resulted in successful experiences in various parts of Brazil, with the advance in the articulation between mental health and primary care, with more than 56% of the teams carrying out actions in this area^12^, such as promotion of MH actions related to matrix support (AM), networking, diversity of practices, and social participation. Matrix support could increase the resolution and effectiveness of the actions of the FHS, with joint action between specialists and professionals from the teams in the field. Remarkable experiences^13,14^ enhanced the collective construction of knowledge and improved communication between workers, users, and managers. Moreover, they expand knowledge about mental health and greater co-responsibility, contributing to the identification and acceptance of cases and the construction of unique therapeutic projects.

Other forms of confrontation underway^9,12^ and are showing results. In Salvador, FHS teams carried out home admission to integrate MH actions to the PHC. In Ribeirão Preto, FHS teams opted for home visits, contributing to postponing psychiatric hospitalization by following up on discharge, guidance to family members, and other efforts to avoid the medicalization of psychological distress.

The MH actions developed in PHC contribute to the transformations in the psychiatric care paradigm, determining the deconstruction of the historical distance between excluding psychiatric practices and primary care. Thus, they reorient the model of care provided to people with mental suffering with community devices and are configured as a reality for the implementation of the National Policy on Mental Health^12^.

This set of significant challenges and experiences indicates that the inclusion of MH actions in PHC constitutes a wide field of possibilities and complex issues. This study seeks to contribute to the assessment of this assertion. It intends to analyze whether primary care teams that have better-structured PHC attributes could offer better assistance in the area of Mental Health.

## METHODS

Cross-sectional study based on data from the second cycle of the PMAQ-AB, coming about in 2013 and 2014 and involving 31,587 Primary Care Teams (EqAB) from Brazil as a whole. Data are shown in module II and relate to the external assessment and to the teamwork process, wherein questions are answered by the coordinator and contain 750 questions. The PMAQ-AB data collection was coordinated by a group of researchers from universities and research institutions responsible for the external evaluation. Researchers trained and accompanied the field interviewers and data collection supervisors.

31,587 EqAB took part in the study. However, 1,809 teams were excluded for various reasons: 713 did not participate in the entire evaluation cycle; 353 did not comply with the commitments assumed in the contract, and 743 obtained a zero grade. The final universe analyzed included 29,778 teams.

From these data, two typologies were elaborated: PHC structuring, based on its essential attributes (independent variable); and the quality of care provided in the area of Mental Health (dependent variable). In the first typology, researchers used the Delphi technique to agree on the questions that could compose each attribute. All items of the questionnaire were condensed into two hundred and five (205) questions and, thus, they were sent to five researchers with a doctoral degree in Public Health, authors of publications in national reference journals on the subject and linked to higher education institutions in different states. The goal was for these scientists to indicate which attribute each question referred to. This process excluded 70 questions: the judges defined 35 of them as “not applicable” and the other 35 mentioned the MH, being eliminated to avoid collinearity. After four rounds, experts achieved a consensus, allowing the assessment of each attribute of PHC.

The Box summarizes the relationship between each Essential Attribute of the PHC and the contents of the questions used in the PMAQ-AB, according to the consensus defined by experts.

For the typology of Quality of care provided in mental health, 35 questions were identified in form II PMAQ-AB, which include reception, consultation, follow-up, test requests, prescription of psychotropic drugs, referral to specialized services, and the multidisciplinary approach of NASF workers toward teams. More detailed questions were also asked about the professionals’ work process, involving treatment, promotion, prevention, harm reduction, and health rehabilitation for people with psychological distress and users of alcohol and other drugs. After the elaboration of typologies, researchers added questions and the categorized indexes (Box).

**Box:** List of PMAQ-AB contents related to each essential attribute of PHC – expert consensus.

Essential attribute	Condensed content of the questions used in the PMAQ
First contact	- Structuring the area covered by the team;
	- Attendance to spontaneous demand;
	- Home visits;
	- Activities related to scheduling appointments;
	- Reception and waiting time at the unit;
	- Agenda organization;
	- Attention to traditional communities; seated; rural population;
	- Existence of transport.
Longitudinality	- Telehealth management;
	- Medical records;
	- Forms and registration of actions performed;
	- User satisfaction;
	- Communication channels with the population;
	- Social control.
Integrality	- Permanent education and training;
	- Planning activities;
	- Availability of information;
	- Institutional support;
	- Execution of planned activities;
	- Articulation of work with local possibilities;
	- Structure, inputs and instrumentalization of actions;
	- Routing of demands;
	- Team work process;
	- Attention to smoking, tuberculosis and leprosy;
	- PICS and bodily practices;
	- Health promotion and education;
	- *Bolsa Família* and *Saúde na Escola* Programs;
	- Home care;
	- NASF Structure.
Care coordinations	- Articulation with HEIs;
	- Monitoring and control of the developed actions;
	- Results evaluation;
	- Support for complexities;
	- Matrix support;
	- Counter-referral;
	- NASF Functioning;
	- Assessment instruments;
	- Management of serious cases;
	- Control and evaluation of developed programs.

PMAQ: *Programa de Melhoria do Acesso e da Qualidade* (Access and Quality Improvement Program). PICS: *Práticas Integrativas Complementares em Saúde* (Complementary Integrative Practices in Health). NASF: *Núcleo Ampliado de Saúde da Família* (Expanded Family Health Nucleus). HEI = Higher Education Institution

The two typologies (attributes and mental health) had their indexes categorized into three scenarios to express the low (0–32.99%), medium (33–65.99%), and high (66–100%) levels. The essential attributes expressed in its category are Integrality, Coordination, Longitudinality, and First Contact, in addition to a general variable, PHC, resulting from the sum of the other four that make up this typology. For measuring purposes, researchers recoded the 18 polytomous variables of the second typology as dichotomous in their answers (1 = yes and 0 = no/don’t know/not applicable/no answer).

In the descriptive analysis, the EqAB were distributed according to the structure of mental health care, the essential and general attributes of the PHC, and the classification by regions. We performed multinomial logistic regression analyzes to investigate the associations, sustaining the healthcare quality provided in MH as the dependent variable and the PHC attributes as the independent variable. The scenario of Low Structuring of MH was assumed as a reference category. Therefore, categories of typologies were dichotomized into low and medium/high.

The magnitude of the associations was represented by the odds ratio Odds Ratio (OR), with a respective confidence interval of 95% (95%CI) and significance level of 5% (p ≤ 0.05). We used the SPSS Statistics 20 program to perform statistical analyses.

The study complies with the standards and regulatory guidelines for research involving human beings of Resolution 466/2012. The *Comitê de Ética em Pesquisa da Universidade Federal de Minas Gerais* (Research Ethics Committee of the Federal University of Minas Gerais) approved this research, Registration 28,804, 5/30/2012.

## RESULTS

Considering the distribution of Mental Health care (Table 1) by levels and regions, we observed that 52.1% of the teams are at the medium level, 29.2% at the low level and a small portion at the high level 18.7%.

**Table 1:** Distribution of quality of mental healthcare by levels and regions, Brazil – PMAQ-AB 2014.

Regions	Levels					
	Low		Average		High	
	n	%	n	%	n	%
South	1,118	24.8	2,370	52.6	1,021	22.6
Southeast	2,288	22.7	5,416	53.6	2,396	23.7
Midwest	933	41.6	1,082	48.3	226	10.1
Region	1,142	52.9	867	40.1	151	7
Northeast	3,210	29.8	5,774	53.5	1,784	16.6
Total	8,691	29.2	15,509	52.1	5,578	18.7

PMAQ-AB: *Programa de Melhoria do Acesso e da Qualidade da Atenção Básica* (Access and Quality Improvement Program in Primary Care).

The Southeast region had the highest number of high-level teams (23.7%) and the lowest number of low-level teams (23.7%), whereas the North region had the opposite indices: 52.9% of low-level and 7% high level.

Considering PHC structuring by levels and regions (Table 2), most teams are located at the average level of PHC structuring (86.52%), 7.5% are at the low level, and only 5.93% are at the high level of structuring.

**Table 2:** Distribution of the primary healthcare structuring by regions and levels, Brazil – PMAQ-AB 2014.

Regions	Levels					
	Low		Averrage		High	
	n %		n %		n %	
South	364	8.1	3,860	85.6	285	6.3
Southeast	670	6.6	8,668	85.8	762	7.5
Midwest	209	9.3	1,961	87.5	71	3.2
Region	365	16.9	1,762	81.6	33	1.5
Northeast	638	5.9	9,514	88.4	616	5.7
Total	2,246	7.55	25,765	86.52	1,767	5.93

PMAQ-AB: *Programa de Melhoria do Acesso e da Qualidade da Atenção Básica* (Access and Quality Improvement Program in Primary Care).

For the regional distribution, the Southeast region once again had the highest number of high-level teams (7.5%), while the Northeast region had the lowest EqAB index at the low level (5.9%). The North Region stands out negatively for presenting the worst rates at all levels, with the largest number of low-level teams (16.9%) and the smallest number of high-level teams (1.5%).

In the distribution of the PHC structuring according to the essential attributes, by levels (Table 3), the Longitudinality attribute stands out, which presents 36% of the high-level EqAB. On the other hand, the Coordination attribute has the worst rates (28% low level and 0.7% high level);

**Table 3:** Distribution of the primary healthcare structuring, according to essential attributes by levels, Brazil – PMAQ-AB 2014.

Essential PHC Attributes	Levels					
	Low		Average		High	
	n %		n %		n %	
Integrality	2,392	8.0	24,130	81.0	3,256	10.9
Coordination	8,344	28.0	21,230	71.3	204	0.7
Longitudinality	1,253	4.2	17,814	59.8	10,711	36.0
First contact	4,008	13.5	25,069	84.2	701	2.4

PMAQ-AB: *Programa de Melhoria do Acesso e da Qualidade da Atenção Básica* (Access and Quality Improvement Program in Primary Care).

In the association analysis (Table 4) between the quality of care provided in mental health and the structuring of primary care, based on the essential attributes by levels, we observed a positive association in medium and high-level PHC structuring scenarios. In the medium structuring scenario, the chance of achieving a higher quality of care in the mental health area increases (OR = 2.193). This result becomes significantly higher (OR = 14.742) when the scenario is highly structured. The better the PHC structure, the better chances of assistance levels increasing to fourteen times greater. Positive associations in the highly structured PHC scenario were found for all attributes (completeness OR = 3.21; coordination OR = 2.31; longitudinality OR = 2.25; first contact OR = 1.55). Finally, for the medium structuring scenario, only the coordination, and first contact attributes showed a positive association.

**Table 4:** Quality of MH care and PHC structuring by essential attributes and general indicator, Brazil – PMAQ-AB 2014.

PCH structuring	Quality of MH care				
	Low		Average	High	
	OR (IC95%)	p	OR (95%CI)	p	OR (95%CI)
Integrality	1	0.32	0.91 (0.75–1.10)	0.00	3.21 (2.60–3.96)
Coordination	1	0.00	1.42 (1.27–1.59)	0.00	2.31(1.69–3.16)
Longitudinality	1	0.87	0.98 (0.77–1.25)	0.00	2.25 (1.74–2.90)
First contact	1	0.00	1.28 (1.11–1.49)	0.00	1.55 (1.23–1.95)
General indicator	1	0.00	2.19 (1.85–2.59)	0.00	14.74 (12.18–7.83)

PMAQ-AB: *Programa de Melhoria do Acesso e da Qualidade da Atenção Básica* (Access and Quality Improvement Program in Primary Care); PHC: Primary Health Care; SM: mental health; OR: odds ratio; 95%CI: 95% confidence interval.

## DISCUSSION

This study demonstrates that Brazil has a long way to reach a high level of mental health care regarding PHC. Most of the teams are at an average level of mental care quality and almost a third of them have a poor level of quality, differing significantly from the general framework of PHC structuring in Brazil.

The number of teams in the worst quality scenario of MH (29.2%) tripled in comparison with PHC structuring. These results show problems highlighted by other studies that point to the ongoing process of structuring the PHC, with the challenge of incorporating mental health practices still lacking^7,9^.

Indeed, higher PHC structuring co-occurs with better mental health actions, a reality that some studies focusing on urban centers have also found in structured MH services in network^4,10,15^.

The data showed that the South and Southeast regions have better results in mental health care, while the North and Center-West regions have the worst results. This suggests that those regions are structured in different ways to include the care of MH in the PHC^7,16^.

The analysis of PHC structuring degree indicates that few teams have a high degree of structuring and a significant majority are at the medium level, corroborating the results of other studies in face of percentage differences^15,17^.

Regarding regions, it is observed that the relationship of regional differences is maintained both for the quality of care in MH and for the structuring of the PHC, indicating that the northern region had the worst results. Socioeconomic factors, problems in retaining professionals, and poor training processes are factors that contribute to this unfavorable scenario^15,16^.

In general, national studies^7,9,17,18^ demonstrate a significant improvement in the structuring of PHC over time. This process enables the insertion of mental health actions at the primary level, promoting improved care

Factors such as facing the challenges of psychiatric reform, the creation and expansion of services, the institution of RAPS, expansion, and definition of PHC as a priority for care, and having a wide range of substitute services are significant advances for mental health and primary care^7,19,20^. However, it is still necessary to improve the insertion of MH actions in PHC.

There are controversies in the various studies on the structuring of attributes in PHC. Research using PCAtool highlights that first contact is one of the most fragile attributes of PHC^21,22^. However, another study, using data from the PMAQ, and FHS (units and users), pointed out longitudinality and coordination as the worst evaluated attributes^19^. This study, however, shows that the attributes act differently in high and low PHC structuring scenarios. The highest number of FHS in the best scenario has the structured longitudinality attribute. For the worst scenario, the coordination attribute is the one that needs to be structured.

The results of the analyzes of the association between the quality of care and the structuring of the PHC demonstrate a positive association that increases by 14 times the chance of the PHC to provide better services in mental health when the EqAB are better structured. In this sense, teams must prepare themselves to absorb care in MH, with professional training, changes in the teams’ work processes, and adaptation of the physical structure to face this great challenge^7^.

We also stress that the inclusion of mental health actions is also resisted by professionals, who need to incorporate the principles of the substitute model, whence the EqAB prioritize mental disorders in the same way they do with other diseases^4,23,24^. This research finds out how much the MH area still needs to advance in its organization process, considering the Brazilian primary care and focusing on mental health in the PHC structuring process.

Although adherence to the 2nd cycle of the PMAQ-AB was quite high, the analysis of only the teams that voluntarily joined may overestimate some results, which presents a limitation for this study. The link of the PMAQ-AB with financial resources to support the program can also compromise the results found. The PMAQ-AB data collection instrument also presents limitations in the formulation of some questions in the mental health area, such as in the characterization of investigated subgroups (users of drugs and psychotropic medications) and of some offered actions. Still, other aspects related to group care, prevention, and mental health promotion actions are not adequately structured in the PMAQ-AB. Therefore, mental health care is underdeveloped, even though understood as part of the scope of ABS actions. Despite these limits, the data from the PMAQ-AB allow a view of how the area is structured in PHC in Brazil.

## CONCLUSIONS

The quality of care in MH in PHC in Brazil still has a long way to go, more than a third of EqAB have poor quality of structuring actions. Mental health care does not follow the structuring of PHC in a linear way, requiring additional efforts for its implementation. We observed that comprehensiveness and coordination are attributes that most contribute to a better offer of good quality care.

The mental health movement has structured the care network centered on substitute equipment. Nowadays, developing MH actions in PHC becomes even more important, because mental health policies are facing setbacks that might be catastrophic. Thus, the consideration of such aspects can significantly contribute to the realization of mental health care with quality and excellence.
