# Supplementary material for: Why does mental health care not follow the structuring of primary care?
Source: Rev Saude Publica. 2021 Nov 23;55:99. doi: 10.11606/s1518-8787.2021055003344 (PMC8647984; doi:10.11606/s1518-8787.2021055003344)
Supplement: Translation

# Translation: Por que a assistência em saúde mental não acompanha a estruturação da atenção primária?

**Authors:** Antonio Moacir de Jesus Lima, Eli Iola Gurgel Andrade, Antonio Thomaz Gonzaga da Matta Machado, Alainer de Fátima dos Santos

**Affiliations:** I Diamantina MG Brasil Universidade Federal dos Vales do Jequitinhonha e Mucuri. Faculdade de Ciências Biológicas e da Saúde. Departamento de Enfermagem. Diamantina, MG, Brasil; II Belo Horizonte MG Brasil Universidade Federal de Minas Gerais. Faculdade de Medicina. Programa de Pós-Graduação em Saúde Pública. Belo Horizonte, MG, Brasil; III Belo Horizonte MG Brasil Universidade Federal de Minas Gerais. Faculdade de Medicina. Departamento de Medicina Preventiva e Social. Belo Horizonte, MG, Brasil

**Keywords:** Assistência à Saúde Mental, Atenção Primária à Saúde, Qualidade da Assistência à Saúde, Administração de Serviços de Saúde

## Abstract

**OBJETIVO:**

Verificar se equipes de atenção básica que possuem atributos mais bem estruturados da atenção primária à saúde (APS) conseguem oferecer melhor assistência em saúde mental (SM).

**MÉTODOS:**

Estudo transversal realizado a partir dos dados da avaliação externa do segundo ciclo do Programa de Melhoria do Acesso e da Qualidade da Atenção Básica (PMAQ-AB), envolvendo 31.587 equipes de atenção básica, entre 2013 e 2014. Foram construídas duas tipologias: qualidade da assistência em saúde mental (variável dependente) e estruturação da APS segundo atributos essenciais (variável independente). Para a construção da tipologia de saúde mental, foram identificados conteúdos sobre o tema no módulo II do PMAQ e realizados somatórios das perguntas para a categorização dos índices. Para a estruturação da APS segundo atributos, utilizou-se técnica Delphi para consensualidade em quatro rodadas referendadas por especialistas. Com análises de regressão logística multinomial, verificou-se associação entre as tipologias e identificou-se qual atributo mais contribuía para qualidade da atenção em saúde mental.

**RESULTADOS:**

Verificou-se que 29,2% das equipes encontram-se em um nível baixo de qualidade em assistência à SM, enquanto 7,5% das equipes apresentam um nível baixo de estruturação da APS segundo atributos essenciais. Diferenças regionais são mantidas, tanto para estruturação da APS quanto para a qualidade da assistência à saúde mental. Evidenciou-se uma chance maior de realizar uma assistência em SM com melhor qualidade quando a APS está mais bem estruturada em nível alto (OR = 14,74) e em nível médio (OR = 2,193). Alto nível de Integralidade está associado a alto nível de Qualidade da Assistência em SM (OR = 3,21).

**CONCLUSÕES:**

Os resultados indicam que há predomínio de baixos níveis de qualidade da assistência à saúde mental, em descompasso com o processo de estruturação da APS, considerando seus atributos essenciais.

## INTRODUÇÃO

Em todo o mundo, os transtornos mentais representam uma parcela elevada dos registros de doenças, só nas Américas, um estudo^1^ revelou prevalência de 17%, sendo 22,5% das doenças na América do Norte e 14,8% em toda América Latina. No Brasil, os transtornos mentais também apresentam taxa considerável 29,5%, tendo a ansiedade, 19,9%, e os transtornos de humor, 11%, como os mais prevalentes, seguidos pelos transtornos de controle de impulso, 4,2%, e outros, decorrentes do uso de substâncias psicoativas, 3,6%, números que fizeram o país pular da sexta para a terceira posição^2^ no estudo de Carga Global de Doença (*Global Burden of Disease* – GBD), entre 1990 e 2015.

A triagem para detecção e tratamento precoce realizada na atenção primária em saúde (APS) pode melhorar a qualidade de vida dos pacientes, ajudar no melhor uso dos investimentos em assistência à saúde e reduzir significativamente as complicações e comorbidades médicas^3^. Assim, a saúde mental (SM) deve ser entendida como um campo de intervenção prioritário para as equipes da Estratégia de Saúde da Família (ESF), devido a sua atuação próxima à comunidade, não apenas nas unidades de saúde, como também no espaço social em que vivem e circulam os usuários.

Contudo, a inserção da saúde mental na APS encontra dificuldades em alguns aspectos, como demonstram diversos estudos. Um desses entraves está na formação profissional, pois os trabalhadores desenvolviam suas ações pautados no modelo biológico, com intervenções centradas na consulta clínica, na medicalização e no diagnóstico, visando apenas à remissão dos sintomas^4^. Outros estudos reforçam essa percepção e apontam para a ausência de corresponsabilização em encaminhamentos desnecessários e uma importante fragilidade na capacidade de identificação de casos por parte dos profissionais da ESF^5,6^. Mais recentemente, outro estudo apontou que mais de 60% dos profissionais da APS afirmaram sentir-se despreparados para acolherem as demandas de saúde mental^7^.

Outro empecilho para essa inserção vincula-se ao processo de trabalho das equipes, pois, ainda que algumas práticas sejam inovadoras, ainda não se identificam as mudanças necessárias para humanização do cuidado às pessoas com sofrimento psíquico^8^. Contudo, esse processo ainda está em fase de implementação e, portanto, necessita de ampliação do acesso, por meio da melhoria da assistência^5^.

Essa ampliação do acesso passa também por questões de infraestrutura, tanto das unidades básicas e quanto de recursos humanos, e de desestruturação da rede de atenção, que ainda mantém uma organização hierárquica e uma dinâmica comunicacional pautada em protocolos técnicos de saúde^9^. Em Salvador, Bahia, por exemplo, um estudo apontou uma dependência da Rede de Atenção Psicossocial (RAPS) do hospital psiquiátrico, apontando para a necessidade de ações que promovam o fortalecimento da rede, sua articulação e desinstitucionalização do Centro de Atenção Psicossocial (CAPS)^10^. Em Fortaleza, Ceará, evidenciou-se a prática de medicalização aos transtornos mentais como forma de mitigar as dificuldades de acesso ao CAPS enfrentadas pelos pacientes^11^.

No entanto o engajamento dos trabalhadores tem resultado em experiências bem-sucedidas em diversas partes do Brasil, contatando-se o avanço na articulação entre a saúde mental e a atenção primária, com mais de 56% das equipes realizando ações nessa área^12^, como de promoção de ações de SM relacionadas ao apoio matricial (AM), ao trabalho em rede, diversidade de práticas e participação social. O matriciamento poderia aumentar a resolutividade e a efetividade das ações da ESF, com a atuação conjunta entre especialistas e profissionais das equipes no território. As experiências conhecidas^13,14^ potencializaram a construção coletiva de saberes e melhoria na comunicação entre os trabalhadores, os usuários e os gestores, além de ampliar os conhecimentos sobre saúde mental e maior corresponsabilização, o que contribui para a identificação e o acolhimento dos casos e construção de projetos terapêuticos singulares.

Outras formas de enfrentamento também estão em curso^9,12^ e mostrando resultados, por exemplo, em Salvador, onde tem sido praticada a Internação Domiciliar, realizada pelas equipes da ESF para integrar as ações de SM na APS, e em Ribeirão Preto, onde têm-se optado pelas Visitas Domiciliares, que contribuem para postergar a internação psiquiátrica por meio do acompanhamento dos egressos, orientações aos familiares e demais esforços para evitar a medicalização do sofrimento psíquico.

As ações de SM desenvolvidas na APS contribuem para as transformações no paradigma da assistência psiquiátrica, determinando a desconstrução do histórico distanciamento entre as práticas psiquiátricas excludentes e a atenção primária. Assim, reorientam o modelo da assistência prestada aos portadores de sofrimento mental com dispositivos comunitários e se configuram como uma realidade para implementação da Política Nacional de Saúde Mental^12^.

Esse conjunto de desafios e experiências significativas indica que a inserção de ações de SM na APS constitui um amplo campo de possibilidades e de complexas questões. Este estudo busca contribuir para avaliar essa inserção e objetivou analisar se as equipes de atenção básica que apresentam atributos da APS mais bem estruturados conseguem oferecer melhor assistência na área da Saúde Mental.

## MÉTODOS

Estudo transversal realizado a partir dos dados do segundo ciclo do PMAQ-AB, ocorrido nos anos de 2013 e 2014, envolvendo 31.587 Equipes de Atenção Básica (EqAB), de todas as regiões do país. Para esse estudo, foram utilizados os dados contidos no módulo II, referente à avaliação externa e relacionado ao processo de trabalho das equipes, em que as perguntas são respondidas pelo coordenador e contém 750 perguntas. A coleta de dados do PMAQ-AB foi coordenada por um grupo de pesquisadores de universidades e instituições de pesquisa responsáveis pela avaliação externa, que treinou e acompanhou os entrevistadores de campo e supervisores da coleta de dados.

Fizeram parte do estudo 31.587 EqAB, no entanto, foram excluídas 1.809 equipes por motivos diversos: 713 não participaram de todo o ciclo de avaliação; 353 não cumpriram com os compromissos assumidos na contratualização e 743 obtiveram nota zero. O universo final analisado contemplou 29.778 equipes.

A partir desses dados, foram elaboradas duas tipologias: Estruturação da APS, a partir dos seus atributos essenciais (variável independente); e Qualidade da Assistência prestada na área da Saúde Mental (variável dependente). Na primeira tipologia utilizou-se a técnica Delphi para a consensualização das perguntas que poderiam compor cada atributo. Todos os itens do questionário foram condensados em duzentas e cinco (205) perguntas e, assim, foram enviadas para cinco pesquisadores com titulação de doutorado em Saúde Coletiva, autores de publicações em periódicos de referência nacional para o tema e vinculados a instituições de ensino superior em diferentes estados. O objetivo era que esses cientistas indicassem a qual atributo cada pergunta se referia. Esse processo excluiu 70 perguntas: 35 delas, os juízes definiram como “não se aplica” e as outras 35 faziam referência à SM e foram eliminadas para evitar colinearidade. Assim, foi possível construir um consenso, após quatro rodadas, permitindo isoladamente a avaliação de cada um dos quatro atributos essenciais da APS.

O Quadro expressa de forma sintética a relação entre cada Atributo Essencial da APS e os conteúdos das questões utilizadas no PMAQ-AB, de acordo com o consenso definido pelos especialistas.

Para a tipologia da Qualidade da atenção prestada em saúde mental, foram identificadas 35 perguntas no formulário II PMAQ-AB, quecontemplam o acolhimento, a consulta, o acompanhamento, a solicitação de exames, a prescrição de psicotrópicos, o encaminhamento para os serviços especializados e a abordagem multiprofissional dos trabalhadores do NASF com as equipes. Também foram realizadas perguntas mais detalhadas sobre o processo de trabalho dos profissionais,, envolvendo tratamento, promoção, prevenção, redução de danos e a reabilitação da saúde aos portadores de sofrimento psíquico e aos usuários de álcool e outras drogas. Construídas as duas tipologias, as questões foram somadas e os índices, categorizados (Quadro).

**Quadro:** Lista de conteúdos do PMAQ-AB relacionados a cada atributo essencial da APS – consenso dos especialistas.

Atributo essencial	Conteúdo condensado das questões utilizadas no PMAQ
Primeiro contato	- Estruturação da área de abrangência da equipe;
	- Atendimento da demanda espontânea;
	- Visitas domiciliares;
	- Atividades relativas ao agendamento de consultas;
	- Acolhimento e tempo de espera na unidade;
	- Organização da agenda;
	- Atenção às comunidades tradicionais; assentados; população rural;
	- Existência de transporte.
Longitudinalidade	- Manejo do telessaúde;
	- Prontuários;
	- Formulários e registro das ações realizadas;
	- Satisfação do usuário;
	- Canais de comunicação com a população;
	- Controle social.
Integralidade	- Educação permanente e capacitações;
	- Atividades de planejamento;
	- Disponibilidade de informações;
	- Apoio institucional;
	- Execução das atividades planejadas;
	- Articulação do trabalho com as possibilidades locais;
	- Estrutura, insumos e instrumentalização das ações;
	- Encaminhamento de demandas;
	- Processo de trabalho da equipe;
	- Atenção ao tabagismo, tuberculose e hanseníase;
	- PICS e práticas corporais;
	- Promoção e educação em saúde;
	- Programas Bolsa Família e Saúde na Escola;
	- Cuidado domiciliar;
	- Estrutura do NASF.
Coordenação do cuidado	- Articulação com IES;
	- Monitoramento e Controle das ações desenvolvidas;
	- Avaliação de resultados;
	- Suporte para complexidades;
	- Apoio matricial;
	- Contrarreferência;
	- Funcionamento do NASF;
	- Instrumentos de avaliação;
	- Gestão dos casos graves;
	- Controle e avaliação dos programas desenvolvidos.

PMAQ: Programa de Melhoria do Acesso e da Qualidade. PICS: Práticas Integrativas Complementares em Saúde. NASF: Núcleo Ampliado de Saúde da Família. IES: instituição de ensino superior.

As duas tipologias (atributos e saúde mental) tiveram seus índices categorizados em três cenários, para expressar os níveis baixo (0–32,99%), médio (33–65,99%) e alto (66–100%). Os atributos essenciais expressos em sua categoria são Integralidade, Coordenalção, Longitudinalidade e Primeiro Contato, além ainda de uma variável geral, a APS, resultante da soma das outra quatro que compõem essa tipologia. Para efeito de pontuação, as 18 variáveis politômicas da segunda tipologia foram recodificadas como dicotômicas em suas respostas (1 = sim e 0 = não/não sabe/não se aplica/não respondeu).

Na análise descritiva, as EqAB foram distribuídas conforme a estruturação da assistência em saúde mental, os atributos essenciais e geral da APS, além da classificação por regiões. Para investigar as associações, foram realizadas análises de regressão logística multinomial, tendo como variável dependente a qualidade da assistência prestada em SM e, como variável independente, os atributos da APS. O cenário de Baixa Estruturação de SM foi assumido como categoria de referência e, portanto, as categorias das tipologias foram dicotomizadas em baixo e médio/alto.

A magnitude das associações foi representada pela razão de chances *odds ratio* (OR), com respectivo Intervalo de Confiança de 95% (IC95%) e nível de significância de 5% (p ≤ 0,05). Utilizou-se o programa SPSS Statistics 20 para realizar todas as análises estatísticas.

O estudo cumpre as normas e diretrizes regulamentadoras de pesquisa envolvendo seres humanos da Resolução 466/2012, com aprovação do Comitê de Ética em Pesquisa da Universidade Federal de Minas Gerais, com o Registro 28.804, em 30/5/2012.

## RESULTADOS

Na distribuição da qualidade dos cuidados em Saúde Mental (Tabela 1), por níveis e regiões, observa-se que 52,1% das equipes se encontram no nível médio, 29,2% no nível baixo e uma pequena parcela no nível alto 18,7%.

**Tabela 1:** Distribuição da qualidade de atenção em saúde mental, por níveis e regiões, Brasil – PMAQ-AB 2014.

Regiões	Níveis					
	Baixo		Médio		Alto	
	n	%	n	%	n	%
Sul	1.118	24,8	2.370	52,6	1.021	22,6
Sudeste	2.288	22,7	5.416	53,6	2.396	23,7
Centro-Oeste	933	41,6	1.082	48,3	226	10,1
Norte	1.142	52,9	867	40,1	151	7
Nordeste	3.210	29,8	5.774	53,5	1.784	16,6
Total	8.691	29,2	15.509	52,1	5.578	18,7

PMAQ-AB: Programa de Melhoria do Acesso e da Qualidade da Atenção Básica.

A região Sudeste apresentou maior número de equipes de nível alto (23,7%) e o menor número de nível baixo (23,7%), enquanto a região Norte tem os índices inversos: 52,9% das equipes de nível baixo e 7% de nível alto.

Quando a distribuição é feita por estruturação da APS, níveis e regiões (Tabela 2), a maioria das equipes situa-se no nível médio de estruturação da APS (86,52%), 7,5% estão no nível baixo e apenas 5,93% se encontram no nível alto de estruturação.

**Tabela 2:** Distribuição da estruturação da atenção primária à saúde, por regiões e níveis, Brasil – PMAQ-AB 2014.

Regiões	Níveis					
	Baixo		Médio		Alto	
	n %		n %		n %	
Sul	364	8,1	3.860	85,6	285	6,3
Sudeste	670	6,6	8.668	85,8	762	7,5
Centro-Oeste	209	9,3	1.961	87,5	71	3,2
Norte	365	16,9	1.762	81,6	33	1,5
Nordeste	638	5,9	9.514	88,4	616	5,7
Total	2.246	7,55	25.765	86,52	1.767	5,93

PMAQ-AB: Programa de Melhoria do Acesso e da Qualidade da Atenção Básica.

Quanto à distribuição regional, a região Sudeste mais uma vez apresentou o maior número de equipes de alto nível (7,5%), enquanto a região Nordeste apresenta o menor índice de EqAB no nível baixo (5,9%). A Região Norte destaca-se negativamente por apresentar os piores índices em todos os níveis, com o maior número de equipes de nível baixo (16,9%) e menor quantidade de esquipes de nível alto (1,5%).

Na distribuição da estruturação da APS segundo os atributos essenciais, por níveis (Tabela 3), destaca-se o atributo Longitudinalidade destaca-se que apresenta 36% das EqAB de nível alto. Em contrapartida, o atributo Coordenação possui os piores índices (28% de nível baixo e 0,7% de nível alto);

**Tabela 3:** Distribuição da estruturação da atenção primária à saúde, segundo os atributos essenciais, por níveis, Brasil – PMAQ-AB 2014.

Atributos essenciais da APS	Níveis					
	Baixo		Médio		Alto	
	n %		n %		n %	
Integralidade	2.392	8,0	24.130	81,0	3.256	10,9
Coordenação	8.344	28,0	21.230	71,3	204	0,7
Longitudinalidade	1.253	4,2	17.814	59,8	10.711	36,0
Primeiro contato	4.008	13,5	25.069	84,2	701	2,4

PMAQ-AB: Programa de Melhoria do Acesso e da Qualidade da Atenção Básica.

Na análise de associação (Tabela 4) entre a qualidade da assistência prestada em saúde mental e a estruturação da atenção primária, a partir dos atributos essenciais, por níveis, observa-se uma associação positiva em cenários de estruturação da APS de médio e alto nível. No cenário de média estruturação aumenta a chance de alcançar maior qualidade da assistência para a área de saúde mental (OR = 2,193). Esse resultado torna-se expressivamente maior (OR = 14,742) quando o cenário é de alta estruturação, ou seja, quando a APS é melhor estruturada, a chance de melhores níveis de assitência chega a ser quatorze vezes maior. Associações positivas no cenário de alta estruturação da APS foram encontradas em todos os atributos (integralidade OR = 3,21; coordenação OR = 2,31; longitudinalidade OR = 2,25; primeiro contato OR = 1,55). Por fim, para o cenário de média estruturação, apenas os atributos coordenação e primeiro contato apresentaram uma associação positiva.

**Tabela 4:** Qualidade da assistência em SM e estruturação da APS, por atributos essenciais e indicador geral, Brasil – PMAQ-AB 2014.

ESTRUTURAÇÃO da APS	QUALIDADE DA ASSISTÊNCIA SM				
	Baixo		Médio	Alto	
	OR (IC95%)	p	OR (IC95%)	p	OR (IC95%)
Integralidade	1	0,32	0,91 (0,75–1,10)	0,00	3,21 (2,60–3,96)
Coordenação	1	0,00	1,42 (1,27–1,59)	0,00	2,31(1,69–3,16)
Longitudinalidade	1	0,87	0,98 (0,77–1,25)	0,00	2,25 (1,74–2,90)
Primeiro contato	1	0,00	1,28 (1,11–1,49)	0,00	1,55 (1,23–1,95)
Indicador geral	1	0,00	2,19 (1,85–2,59)	0,00	14,74 (12,18–7,83)

PMAQ-AB: Programa de Melhoria do Acesso e da Qualidade da Atenção Básica; APS: Atenção Primária à Saúde; SM: Saúde Mental; OR: odds ratio; IC95%: intervalo de confiança.

## DISCUSSÃO

Este estudo demonstra que há ainda um longo caminho a ser percorrido para se atingir um alto nível de qualidade do cuidado em saúde mental na APS brasileira. A maior parte das equipes encontra-se em nível médio de qualidade do cuidado ofertado e quase um terço delas apresenta nível de qualidade ruim, diferindo bastante do quadro geral de estruturação da APS no Brasil.

O número de equipes que se encontram no pior cenário para a qualidade de assistência à SM (29,2%) é o triplo daquele encontrado para estruturação da APS. Esses resultados constatam problemas evidenciados por outros estudos que apontam o processo em curso de estruturação da APS, faltando ainda enfrentar o desafio da incorporação das práticas de saúde mental^7,9^.

Com efeito, quando há alta estruturação da APS também as ações em saúde mental estão bem estruturadas, realidade que alguns estudos focando centros urbanos também constataram em situações nas quais os serviços de SM já se encontram estruturados em forma de rede^4,10,15^.

Os dados evidenciaram que as regiões Sul e Sudeste possuem resultados melhores em assistência à saúde mental, enquanto as regiões Norte e Centro-Oeste apresentam os piores resultados, o que sugere que as regiões estruturam-se de diferentes formas para inserirem o cuidado de SM na APS^7,16^.

A análise sobre o grau de estruturação da APS constatou que poucas equipes possuem um alto grau de estruturação e uma maioria significativa encontra-se no nível médio, corroborando com os resultados de outros estudos, com diferenças percentuais^15,17^.

Quando se faz análise por regiões, observa-se que se mantém a relação das diferenças regionais tanto para qualidade da assistência em SM quanto para estruturação da APS, indicando que a região norte obteve os piores resultados. Fatores socioeconômicos, problemas para a fixação de profissionais e baixo processo formativo são fatores que contribuem para esse panorama desfavorável^15,16^.

De modo geral, estudos nacionais^7,9,17,18^ demonstram significativa melhoria da estruturação da APS ao longo do tempo e que esse processo possibilita a inserção de ações em saúde mental no nível primário, o que, por sua vez, promove melhora do cuidado

Fatores como o enfrentamento aos desafios da reforma psiquiátrica, a criação e ampliação de serviços, a instituição da RAPS, ampliação e definição da APS como prioritária para o cuidado e ter ampla diversidade de serviços substitutivos são avanços significativos para a saúde mental e a atenção primária^7,19,20^, porém é preciso ainda evoluir na inserção de ações em SM na APS.

Há controvérsias nos diversos estudos sobre a estruturação dos atributos na APS. Pesquisas usando o PCAtool ressaltam que o primeiro contato é um dos atributos mais frágeis da APS^21,22^. No entanto, outro estudo, utilizando dados do PMAQ, com dados das ESF, unidades e usuários, apontou a longitudinalidade e a coordenação como os atributos pior avalidados^19^. Este estudo, porém, evidencia que os atributos atuam de forma diferente em cenários de alta e baixa estruturação da APS. O maior número de ESF no melhor cenário possui o atributo longitudinalidade estruturado; já para o pior cenário, o atributo coordenação é que necessita ser estruturado.

Os resultados das análises de associação entre a qualidade da assistência e a estruturação da APS demonstram uma associação positiva que amplia em 14 vezes a chance de a APS prestar melhores serviços em saúde mental quando as EqAB estão mais bem estruturadas. Nesse sentido, as equipes devem se preparar para absorver o cuidado em SM, com capacitação dos profissionais, mudanças nos processos de trabalho das equipes e adequação de estrutura física para o enfrentamento desse grande desafio^7^.

É preciso levar em consideração que a inserção de ações em saúde mental também sofra resistência por parte dos profissionais, que precisam incorporar os princípios do modelo substitutivo, em que as EqAB priorizam os transtornos mentais da mesma forma que faz com outras doenças^4,23,24^. Ao focalizar a saúde mental no processo de estruturação da APS, esta pesquisa constata o quanto a área de SM ainda precisa avançar em seu processo de organização, acompanhando o fortalecimento da atenção primária no país.

Embora a adesão ao 2º ciclo do PMAQ-AB tenha sido bastante elevada, a análise apenas das equipes que aderiram voluntariamente pode superestimar alguns resultados, o que se apresenta com uma limitação para este estudo. O vínculo do PMAQ-AB com recursos financeiros de suporte ao programa também pode comprometer os resultados encontrados. O instrumento de coleta de dados do PMAQ-AB também apresenta limites na formulação de alguns quesitos na área de saúde mental, como na caracterização de subgrupos investigados (usuários de drogas e de medicamentos psicotrópicos) e de algumas ações ofertadas. Também outros aspectos relativos ao atendimento em grupo, ações de prevenção e de promoção da saúde mental não se encontram adequadamente estruturados no PMAQ-AB. Portanto, a oferta de cuidado em saúde mental, ainda que entendida como parte do escopo de ações da ABS, mostra-se pouco desenvolvida. Apesar desses limites, os dados advindos do PMAQ-AB permitem uma visão de como a área se encontra estruturada na APS no Brasil.

## CONCLUSÕES

A qualidade do cuidado em SM na APS no Brasil ainda tem um longo percurso à frente, mais de um terço das EqAB possui qualidade ruim de estruturação das ações. A atenção à saúde mental não acompanha de forma linear a estruturação da APS, exigindo esforços adicionais para sua implementação. Observou-se que os atributos essenciais que mais contribuem para melhor oferta de cuidados com boa qualidade são integralidade e coordenação.

Historicamente, o movimento de saúde mental tem estruturado a rede de atenção centrada em equipamentos substitutivos. Atualmente, com as políticas de saúde mental enfrentando retrocessos que podem ser catastróficos, inserir ações de SM na APS no Brasil torna-se ainda mais importante. Assim, a consideração de tais aspectos pode contribuir de forma marcante para a efetivação do cuidado em saúde mental com qualidade e excelência.
